# Rest-Activity Rhythms, Their Modulators, and Brain-Clinical Correlates in Opioid Use Disorder

**DOI:** 10.1001/jamanetworkopen.2024.57976

**Published:** 2025-02-04

**Authors:** Rui Zhang, Peter Manza, Sukru Baris Demiral, Dardo Tomasi, Michele-Vera Yonga, Weizheng Yan, Ehsan Shokri-Kojori, Melanie Schwandt, Leah Vines, Diana Sotelo, Christina Lildharrie, Esther Lin, Natasha T. Giddens, Gene-Jack Wang, Nora D. Volkow

**Affiliations:** 1 Laboratory of Neuroimaging, National Institute on Alcohol Abuse and Alcoholism, National Institutes of Health, Bethesda, Maryland; 2 Office of Clinical Director, National Institute on Alcohol Abuse and Alcoholism, National Institutes of Health, Bethesda, Maryland; 3 Department of Psychiatry, University of Wisconsin, Madison

## Abstract

**Importance:**

Sleep and circadian disruptions are highly prevalent in opioid use disorder (OUD) and are a barrier to successful treatment and recovery; yet few objective data are available, especially for individuals in OUD treatment with opioid agonist therapy. If disruptions remain present despite OUD treatment, this information would yield potential new targets for adjunctive therapy.

**Objective:**

To systematically investigate different aspects of rest-activity rhythms (RAR), including sleep, physical activity, circadian rhythmicity, and brain functional correlates in individuals with OUD.

**Design, Setting, and Participants:**

This cross-sectional study conducted from October 12, 2017, through January 11, 2024, recruited participants with OUD from treatment programs or the community in the District of Columbia, Maryland, and Virginia area. Participants included individuals with OUD treated with methadone or buprenorphine, individuals with OUD who remained abstinent without medications, and healthy controls (HCs). Healthy participants were recruited from advertisements. Statistical analyses were conducted between March 1 and May 31, 2024.

**Main Outcomes and Measures:**

In total, 21 RAR features were derived from 1-week actigraphy data, and principal components were used to extract independent RAR components. Modulators and brain and clinical correlates of RAR were also examined.

**Results:**

This study included 73 participants (46 [63%] male; mean [SD] age, 43.5 [11.3] years). Among 42 patients with OUD (16 [38%] female; mean [SD] age, 42.7 [11.4] years), 33 receiving medications for opioid use disorder (MOUD) exhibited greater sleep-wake irregularity than 9 patients without MOUD (mean difference, 0.85 [95% CI, 0.00-1.69]) or 31 age- and sex-matched HCs (11 [36%] female; mean [SD] age, 44.5 [11.3] years; mean difference, 0.75 [95% CI, 0.19-1.31). Among participants receiving MOUD, greater sleep irregularity was associated with longer heroin use history (*r*_26_ = 0.45; *P* = .02) and lower daytime light exposure (*r*_33_ = −0.57; *P* < .001). Compared with HCs, participants with OUD exhibited lower fractional occupancy (percentage of occurrence) in a default mode network–dominated brain state, with individuals experiencing more pronounced sleep-wake irregularities displaying exacerbated impairments (*r*_23_ = −0.55; *P* = .007).

**Conclusions and Relevance:**

Findings of this cross-sectional study showed that sleep irregularity in participants with OUD receiving opioid agonist medications correlated with years of opioid misuse and shorter daylight exposures and was associated with impaired brain state dynamics. These findings suggest that interventions increasing light exposure may improve sleep-wake irregularity and brain functional network dynamics in individuals with OUD receiving opioid agonist medications.

## Key Points

**Questions:**

Are disruptions in rest-activity rhythms (RAR) present in patients with opioid use disorder (OUD), and what factors and brain functions are associated with RAR changes?

**Findings:**

This cross-sectional study of 42 patients with OUD and 31 healthy controls found that patients with OUD who received opioid agonist treatment displayed greater sleep-wake irregularity than healthy controls. Sleep-wake irregularity was associated with years of heroin use, exacerbated impairments in brain state dynamics, and greater light exposure attenuated sleep-wake irregularity in patients with OUD.

**Meaning:**

Findings from this study suggest that light therapy may provide therapeutic benefits as an adjunct to OUD medications.

## Teaser

This cross-sectional study investigates brain state dynamics and rest-activity rhythms, including sleep, physical activity, and circadian rhythmicity, among participants with opioid use disorder who did vs who did not receive treatment with opioid agonist medications.

## Introduction

In 2021, over 75% of the nearly 107 000 drug overdose deaths involved an opioid.^1^ Although medications for opioid use disorder (MOUD) are highly effective in reducing illicit opioid use and overall mortality in patients with opioid use disorder (OUD),^2^ treatment retention rates remain low.^3^ Self-reported sleep disturbances are symptoms that persist during MOUD treatment and are associated with worse clinical outcomes, including withdrawal, psychiatric symptoms, negative affect, and pain, contributing to relapse and dropout.^4^ In contrast, objective sleep and circadian biomarkers have been less studied and there is evidence suggesting a discrepancy between subjective and objective measures in OUD.^5^

Rest-activity rhythm (RAR), referred to as the sleep-wake cycle, characterizes the pattern of a person’s rest and active periods and is strongly modulated by endogenous circadian rhythms. In animals, diurnal activity patterns are altered after continuous morphine administration and during acute withdrawal, including changes in amplitude, day vs night activity levels, and timing of peak activity.^6^ In individuals who misuse heroin, diurnal rhythms of clock gene expression are disrupted during early abstinence along with disrupted diurnal rhythms of certain neuropeptides, including cortisol, adrenocorticotropic hormone, β-endorphin, leptin, and interleukin 2 release. In postmortem brains of individuals with OUD, disrupted circadian rhythms have been identified at the molecular level.^7^ Yet beyond sleep duration, no studies have systematically investigated different components of RAR, for example, phase timing, amplitude and physical activity levels, and rhythm regularity, among individuals receiving treatment with vs without MOUD, to assess whether they differ from healthy controls (HCs) and how these components relate to brain functions. Here we examined various facets of RAR instead of focusing on 1 specific parameter because our previous work suggested that various RAR components (ie, phase timing and physical activity) were associated with drug use through different pathways.^8^ We performed principal component analyses to capture independent RAR components. Given that light affects various aspects of RAR,^9,10^ we further investigated how light exposure was associated with potential RAR changes in individuals with vs without MOUD treatment and compared with HC individuals.

Our previous work revealed altered brain state dynamics in participants with OUD compared with HCs by applying a data-driven clustering approach to identify recurrent coactivation patterns of brain networks (ie, brain states) and their dynamics.^11^ We found that participants with OUD showed lower prevalence in default mode network (DMN)–dominated brain states and higher prevalence in visual network–dominated brain states. In the current study, we tested how RAR components were associated with brain state dynamics and whether they accounted for altered brain function among participants with OUD. We included individuals receiving MOUD, individuals without MOUD treatment who were abstinent from opioid use, and HC individuals to explore how RAR, light exposure, and brain state dynamics differed among them.

## Methods

### Participants

Participants with OUD were recruited for this cross-sectional study from treatment programs and the community, while HCs were recruited through advertisements. Participants who had 1-week actigraphy data were included in the current analyses. All participants were recruited in the Washington metropolitan area (District of Columbia, Maryland, and Virginia) between October 12, 2017, and January 11, 2024, including the pandemic period (January 13, 2020, through May 11, 2023). Self-reported information about race was collected to better characterize the cohorts. Race was categorized into American Indian or Alaska Native, Asian, Black or African American, White, more than 1 race, and other following the National Institutes of Health guideline on race and ethnicity reporting. Participants with OUD met *Diagnostic and Statistical Manual of Mental Disorders* (Fifth Edition) criteria for OUD in their lifetime and had a minimum 5-year history of nonmedical opiate use. Participants with OUD were not excluded if they had an additional substance use disorder, but opioids had to be their preferred drug. On scan and testing days, all participants underwent daily breathalyzer and urine drug screenings (for cocaine, tetrahydrocannabinol, opiates, amphetamine, methamphetamine, and oxycontin), with exclusion criteria applied for positive drug screens in the control group. HCs were matched by age and sex with participants with OUD. Participants in the HC group had no history of substance use disorder or other psychiatric disorder nor current use of prescribed or over-the-counter psychoactive medications. Nicotine use was not an exclusion criterion. Some participants underwent a resting-state functional magnetic resonance imaging (rfMRI) scan. Participants refrained from nicotine or caffeine intake for at least 2 hours before the scan. All participants provided written informed consent prior to participating in this study, which was approved by the Institutional Review Board at the National Institutes of Health. This report followed the Strengthening the Reporting of Observational Studies in Epidemiology (STROBE) reporting guideline for cross-sectional studies.

### Actigraphy Analysis

To capture RAR and sleep-wake patterns, participants wore a triaxial accelerometer (GENEActiv, version 1.1; Activinsights Ltd) on their nondominant wrist continuously for 1 week. For actigraphy analyses, we used both parametric and nonparametric measures, as they offer complementary insights. While parametric measures capture the size, timing, and shape of rhythms,^12^ nonparametric measures are based on raw data counts and do not rely on a priori assumptions about the waveform.^13^ eAppendices 1-3 in Supplement 1 provide detailed methodology, variable definitions, and how missing data were handled. SPSS 22 (IBM Corp) was then used for factor analyses to extract RAR principal components with varimax rotation.

### Light Exposure Analyses

The triaxial accelerometer incorporates a light sensor (silicon photodiode) to record illuminance (lux) of white light (wavelength range of 400-1100 nm, 0-3000 lux, 5-lux resolution, ±10% accuracy at 1000-lux calibration). We calculated the peak lux per 15-minute epoch followed by computing (1) the mean peak lux for the most active 10-hour period (M10); (2) mean light exposure during both daytime and nighttime and defined daytime as the period from 5 am to 9 pm and nighttime from 9 pm to 5 am, instead of relying on biological clock–based definitions for daytime and nighttime light exposure; and (3) mean light timing above a threshold of 500 lux (MLiT^500^). For instance, the MLiT^500^ of 700 minutes indicates that one’s light exposure greater than 500 lux is on average centered around 700 minutes since midnight (12 am).

### Analysis of Brain States And Dynamics

eAppendix 4 in Supplement 1 provides MRI acquisition information, and eAppendix 5 in Supplement 1 gives MRI preprocessing details. We identified brain states (ie, brain coactivation patterns) using a data-driven approach and labeled them based on the cosine similarity with a priori–defined resting-state functional networks. We first parcellated denoised voxel-level data into a 454-node Schaefer atlas (400 cortical regions and 54 subcortical regions)^14,15^ followed by demeaning each region of interest time series. Subsequently, we concatenated demeaned region of interest time series from all participants into a matrix, on which we applied k-means clustering.^16^ We chose k = 5 because additional variance explained by increasing k beyond this level was less than 1% (eFigure 1 and eAppendix 6 in Supplement 1). Centroids of each state were calculated as the mean of the regional activation over all repetition times (TRs) assigned to that state.

Following this analysis, we then analyzed the dynamic characteristics of the identified brain states. Fractional occupancy was defined as the proportion of TRs assigned to each brain state. Dwell time was calculated by determining the mean length of time spent in a brain state. Appearance rate was determined by the total number of times a brain state appeared per minute.

### Statistical Analysis

For comparisons between participants with OUD and HCs and for exploratory analyses comparing OUD treated with methadone vs buprenorphine, independent *t* tests were applied. For comparisons among 3 groups (with or without MOUD treatment and vs HCs), we used 1-way analyses of variance followed by Tukey post hoc tests. Pearson correlations were used to examine correlations, and partial correlations were used to control for age. SPSS 22 (IBM Corp) and RStudio (R Project for Statistical Computing) were used for analyses. The Benjamini-Hochberg (BH) procedure was applied to correct for multiple comparisons (adjusted for the number of RAR components, brain states, or light exposure parameters). Both uncorrected and BH-corrected *P* values as well as effect sizes are reported. Findings with 2-sided values of BH-corrected *P* < .05 are discussed and were considered statistically significant. Statistical analyses were conducted between March 1 and May 31, 2024.

## Results

In the current analyses, we included 73 participants (46 [63%] male; mean [SD] age, 43.5 [11.3] years), 42 with OUD (16 [38%] female and 26 [62%] male; mean [SD] age, 42.7 [11.4] years) and 31 in the HC group (11 [36%] female and 20 [64%] male; mean [SD] age, 44.5 [11.3] years) (Table 1; power analysis in eAppendix 7 in Supplement 1). Data were collected from 36 participants before the pandemic, 29 participants during the pandemic, and 8 participants after the pandemic. Among participants with OUD, 33 received MOUD treatment: 14 with buprenorphine and 19 with methadone. The mean (SD) dose was 89.7 (43.4) mg for the methadone group and 18.4 (6.7) mg for the buprenorphine group. There were participants without MOUD who reported lack of opioid misuse or any medication treatment for at least 3 months prior to the study participation (individuals could have been receiving MOUD before that). There were no smokers in the HC group. Table 1 gives additional demographic and clinical information for participants.

**Table 1.** Demographic and Clinical Characteristics

Characteristic	Group Mean (SD)		*P* value (OUD vs HC)	Group Mean (SD)		*P* value (with vs without MOUD)
	OUD (n = 42)	HC (n = 31)		With MOUD (n = 33)	Without MOUD (n = 9)	
Sex, No. (%)						
Female	16 (38)	11 (36)	.82	12 (36)	4 (44)	.66
Male	26 (62)	20 (64)		21 (64)	5 (56)	
Self-reported race, No. (%)						
Black	13 (31)	14 (45)	.13	10 (30)	3 (33)	.08
White	23 (55)	14 (45)		20 (61)	3 (33)	
Other^a^	6 (14)	3 (10)		3 (9)	3 (33)	
Smoker, No. (%)	36 (86)	0	NA	30 (91)	6 (67)	.06
Age, y	42.7 (11.4)	44.5 (11.3)	.51	44.2 (11.6)	37.2 (9.2)	.11
FTND score^b^	3.55 (2.28)	0	NA	3.84 (2.18)	2.56 (2.46)	.14
AUDIT score^c^	3.00 (4.89)	1.65 (1.08)	.09	2.94 (4.84)	3.22 (5.38)	.88
STAIT score^d^	40.02 (10.15)	26.23 (6.00)	<.001^e^	40.19 (9.80)	39.44 (11.93)	.42
Sleep medication score^f^	0.38 (.73)	0.23 (.67)	0.36	0.42 (.75)	0.22 (.67)	.47
BDI score^g^	9.73 (8.37)	.86 (1.41)	<.001^e^	9.91 (8.91)	9.00 (6.19)	.39
Age of onset (opiates), y	22.2 (7.2)	NA	NA	22.8 (7.8)	20.2 (4.0)	.09
Duration of heroin use, y	10.7 (11.9)	NA	NA	11.5 (12.5)	7.6 (9.3)	.42
Duration of treatment, y	4.0 (5.0)	NA	NA	4.1 (5.5)	4.0 (3.0)	.96
Duration of cocaine use, y	9.3 (10.4)	NA	NA	8.9 (10.7)	10.4 (9.4)	.70
Heroin use past 30 d, No. (%) of users	8 (19)	NA	NA	8 (24)	0	NA
Heroin use past 30 d No. (%) days of use among users	9 (31)	NA	NA	9 (31)	NA	NA
Cocaine use past 30 d, No. (%) of users	6 (14)	NA	NA	6 (18)	0	NA
Cocaine use past 30 d, No. (%) days of use among users	4 (14)	NA	NA	4 (14)	NA	NA
Cannabis use past 30 d, No. (%) of users	13 (31)	NA	NA	12 (36)	1 (11)	NA
Cannabis use past 30 d, No. (%) days of use among users	18 (60)	NA	NA	18 (61)	15 (50)	NA

Abbreviations: AUDIT, Alcohol Use Disorders Identification Test; BDI, Beck Depression Inventory; FTND, Fagerström Test for Nicotine Dependence; HC, healthy control; MOUD, medications for opioid use disorder; NA, not applicable; OUD, opioid use disorder; STAIT, State-Trait Anxiety Inventory.

^a^ Other race included American Indian or Alaska Native, Asian, and more than 1 race.

^b^ FTND scores ranged 0 to 10; 0 to 2 indicates very low dependence, 3 to 4 indicates low dependence, 5 to 7 indicates moderate dependence, and scores above 8 indicate high dependence.

^c^ AUDIT scores ranged from 0 to 40: 0 indicates an abstainer who has never had any problems with alcohol; a score of 1 to 7 suggests low-risk consumption according to World Health Organization guidelines; scores from 8 to 14 suggest hazardous or harmful alcohol consumption; and a score of 15 or more indicates the likelihood of alcohol dependence (moderate to severe alcohol use disorder).

^d^ STAIT scores ranged from 20 to 80, with higher numbers indicating higher anxiety level.

^e^ Independent 2-sided *t* test uncorrected *P* < .05.

^f^ Sleep medication scores from the Pittsburgh Sleep Quality Index questionnaire: 0 represents not during the last month; 1, less than once a week; 2, once or twice a week; and 3, three or more times a week.

^g^ BDI scores ranged from 0 to 63, with higher numbers indicating more severe depression.

### RAR

**Figure 1.**
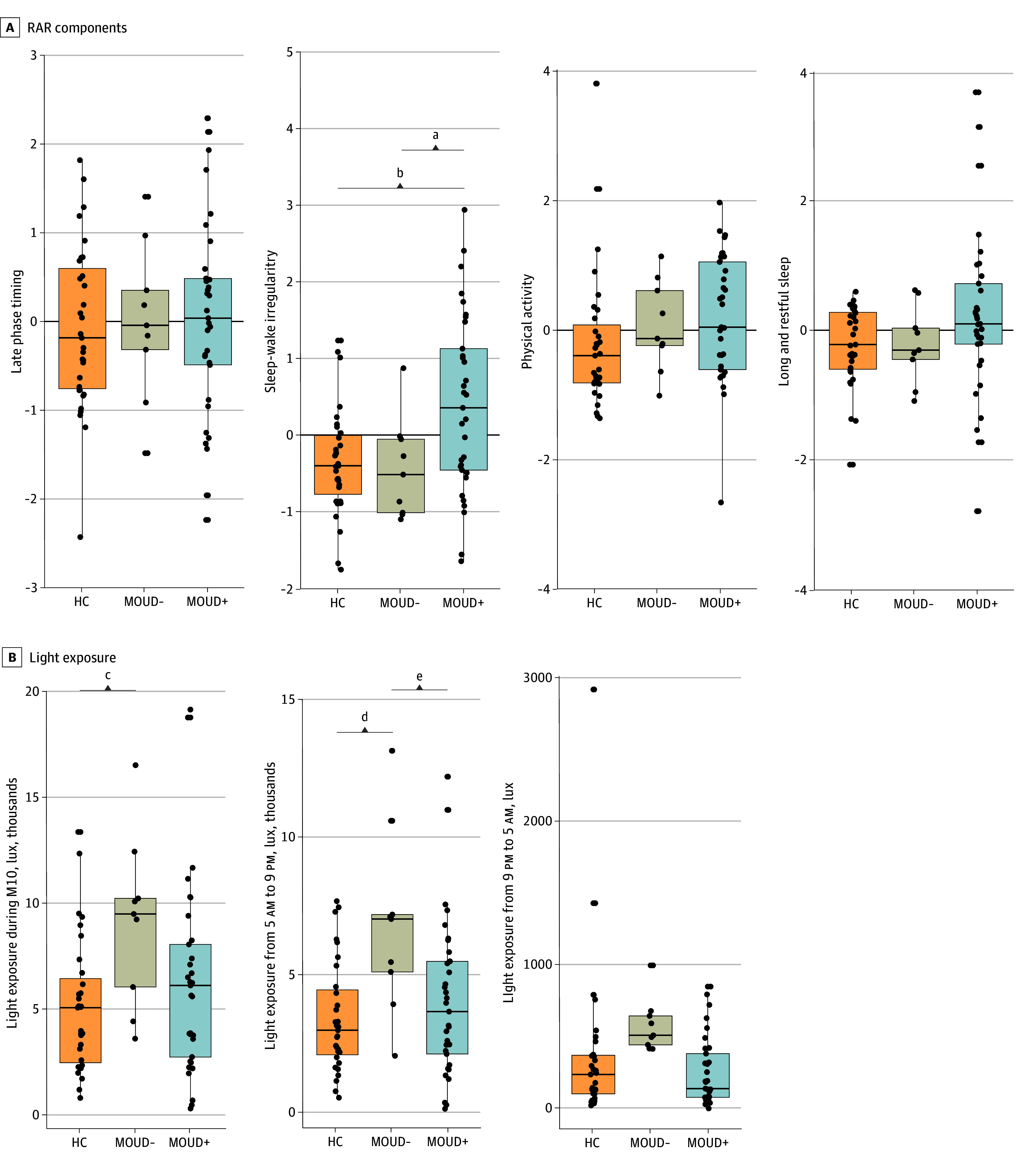
Rest-Activity Rhythm (RAR) Components and Light Exposures Among Participants Receiving (MOUD+) vs Not Receiving (MOUD−) Medications for Treatment of Opioid Use Disorder and vs Participants in the Healthy Control (HC) Group M10 represents the most active 10-h period. Within each box, the center line indicates the median value; the boundaries of the box denote the 25th percentile and the 75th percentile of the distribution. Lower whisker extends to the smallest value in the data that is within 1.5 × IQR below 25th percentile. Upper whisker extends to the largest value in the data that is within 1.5 × IQR above 75th percentile. IQR is the distance between the first and third quartiles. *P* values from 2-sided post hoc tests for the indicate comparison. ^a^*P* = .048. ^b^*P* = .005. ^c^*P* = .02. ^d^*P* = .002. ^e^*P* = .02.

We extracted 4 RAR components from 21 RAR variables that explained 66% of the total variance (eTable in Supplement 1). Table 2 gives descriptive results and comparisons between OUD and HC groups for these 21 RAR variables. We identified 4 RAR components and labeled them based on the greatest loadings of each component: (1) late phase timing, (2) sleep-wake irregularity, (3) physical activity, and (4) long and restful sleep. Participants with MOUD treatment showed greater sleep-wake irregularity than participants in the HC group and participants without MOUD treatment (*F*_2,70_ = 6.24, *P* = .003, BH-corrected *P* = .01; η^2^ = 0.15; post hoc Tukey honestly significant differences [HSD]: group with MOUD treatment vs HC group mean difference, 0.75 [95% CI, 0.19-1.31]; *P *= .005; group with MOUD vs group without MOUD mean difference, 0.85 [95% CI, 0.00-1.69]; *P* = .048), whereas there was no significant difference between participants in the HC group and participants without MOUD in sleep-wake regularity (Tukey HSD *P* = .96) (Figure 1A). The group with OUD and the HC group did not significantly differ in other RAR components (eFigure 2 in Supplement 1). No significant RAR differences were found between patients receiving methadone vs buprenorphine (late phase timing, *t*_31_ = −0.67, *P* = .51; sleep-wake irregularity, *t*_31_ = 1.36, *P* = .18; physical activity, *t*_31_ = −0.58, *P* = .56; long and restful sleep, *t*_31_ = 1.31, *P* = .20). No significant differences were observed for the length of opioid agonist treatment, acute drug use (heroin, cannabis and cocaine use in the last 30 days), pandemic period studied, or employment status (eAppendix 8 in Supplement 1).

**Table 2.** Descriptive Information for 21 RAR Variables and Light Exposure

RAR variable	Group Mean (SD)		*P* value
	Participants with OUD (n = 42)	Participants in the HC group (n = 31)	
Interdaily stability^a^	0.54 (.16)	0.56 (0.13)	.83
Intradaily variation^b^	0.58 (.23)	0.69 (0.18)	.03^c^
Amplitude^d^^,^^e^	2.23 (1.05)	1.68 (0.45)	.003^c^
Alpha^d^	−0.31 (.48)	−0.47 (0.17)	.05
Acrophase, 24-h decimal time^d^	15.4 (2.0)	15.3 (1.6)	.75
Up-mesor, 24-h decimal time^d^	8.2 (2.9)	7.4 (1.5)	.17
Down-mesor, 24-h decimal time^d^	18.2 (8.1)	20.1 (7.2)	.28
Pseudo *F* statistic^d^	248.3 (230.5)	287.6 (213.0)	.52
L5 h, 24-h decimal time	25.5 (1.8)	25.1 (1.6)	.26
L5, milli-gravitational acceleration	5.13 (1.72)	5.17 (1.61)	.70
M10 h, 24-h decimal time	10.0 (1.9)	9.9 (2.0)	.76
M10, milli-gravitational acceleration	51.39 (18.66)	51.68 (33.42)	.97
Daily mean physical activity, milli-gravitational acceleration	30.52 (10.02)	31.03 (16.62)	.87
Sleep duration, h	7.4 (2.3)	7.2 (1.2)	.59
Sleep duration variation, h	2.4 (1.2)	1.8 (0.70)	.02^c^
Sleep onset, 24-h decimal time	23.8 (2.3)	24.0 (1.5)	.58
Sleep onset variation, h	2.4 (1.4)	1.6 (1.0)	.009^c^
Wake time, 24-h decimal time	31.2 (2.0)	31.2 (1.8)	.94
Wake time variation, h	2.2 (1.5)	1.6 (1.0)	.06
Sleep regularity index^f^	42.77 (18.06)	50.97 (11.43)	.02^c^
Sleep regularity index variation	14.98 (9.38)	12.18 (7.04)	.18
M10 mean light exposure, lux	6816.33 (4528.78)	5095.05 (3169.74)	.07
Mean daytime light exposure, lux	4698.35 (3138.75)	3459.69 (2001.99)	.06
Mean nighttime light exposure, lux	322.80 (262.23)	372.65 (555.08)	.61
MLiT^500^, min	773.1 (74.0)	781.2 (77.5)	.65

Abbreviations: HC, healthy control; L5, milli-gravitational acceleration, physical activity level during the least active 5-hour period; M10, milli-gravitational acceleration, physical activity level during the most active 10-hour period; M10 mean light exposure, lux, mean peak lux for the most active 10-hour period; MLiT^500^, mean light timing above a threshold of 500 lux; OUD, opioid use disorder; RAR, rest-activity rhythm.

^a^ Interdaily stability ranged from 0 to 1, with higher values indicating a more consistent daily pattern.

^b^ Intradaily variation ranged from 0 to 2, with higher values indicating greater fragmentation throughout the day and frequent transitions between rest and active periods.

^c^ Independent 2-sided *t* test uncorrected *P* < .05.

^d^ Outputs from parametric modeling.

^e^ Amplitude ranged between 0 and 1, with higher values indicating that the differences between peak and low activity periods are larger and the rhythmicity is more pronounced.

^f^ Sleep regularity index ranged from 0 to 100, with higher values indicating greater regularity of sleep.

### Light Exposure

Participants without MOUD but not participants with MOUD treatment showed greater light exposure during the most active 10 hours (M10; *F*_2,70_ = 3.63, *P* = .03, BH-corrected *P* = .048; η^2^ = 0.09; post hoc Tukey HSD without MOUD group vs HC group, *P* = .02) and daytime light exposure from 5 am to 9 pm than participants in the HC group (*F*_2,70_ = 6.15, *P* = .003, BH-corrected *P* = .009; η^2^ = 0.15; post hoc Tukey HSD without MOUD group vs HC group, *P* = .002; Tukey HSD without MOUD group vs with MOUD group, *P* = .02) (Figure 1B). The OUD and HC groups did not differ in nighttime light exposure (Table 2, eFigure 3 in Supplement 1). There were no differences in light exposure between the group with MOUD treatment and the HC group (post hoc Tukey HSD for M10 *P* = .51, daytime *P* = .53, and nighttime *P* = .30) or between participants with MOUD treated with methadone vs buprenorphine (M10, *t*_31_ = −0.98, *P* = .34; daytime, *t*_31_ = −1.34, *P* = .19; nighttime, *t*_31_ = −1.79, *P* = .08). No group differences were found for timing of light exposure (MLiT^500^, *F*_2,70_ = 0.60, *P* = .55). eAppendix 9 in Supplement 1 describes the associations with daylength.

### Association of Sleep-Wake Irregularity With Low Daytime Light Exposure and Years of Heroin Use

**Figure 2.**
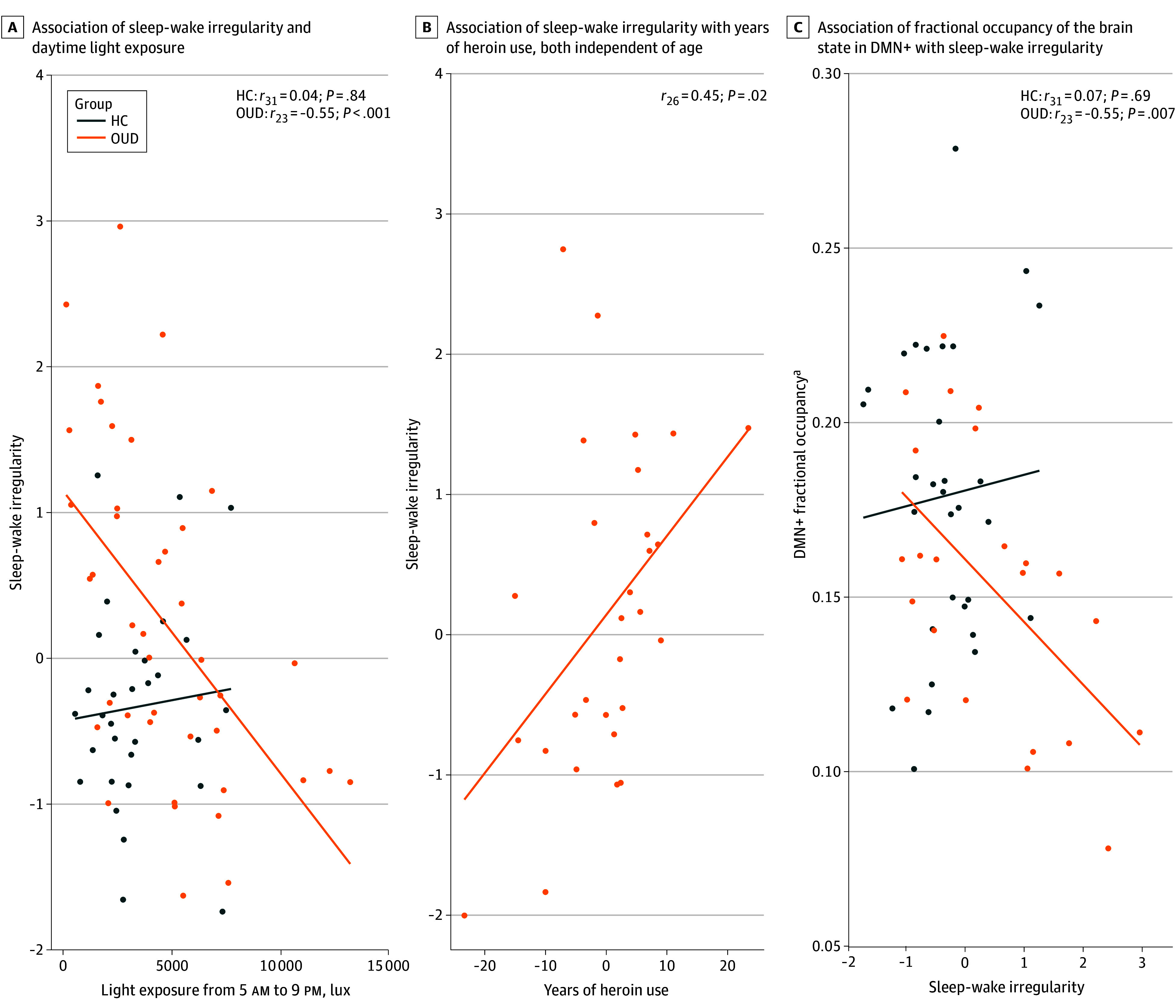
Associations Between Sleep-Wake Irregularity, Light Exposure, Years of Heroin Use, and Brain State Dynamics Each dot represents a single data point. Each line (line of best fit) illustrates the correlation between 2 variables. DMN+ indicates default mode network–dominated brain state, with activity above regional means; HC, healthy control group; OUD, group with opioid use disorder. ^a^Fractional occupancy defined as the proportion of repetition times assigned to each brain state.

We examined the association between sleep-wake irregularity and light exposure in participants with OUD and in the HC group separately. Among participants with OUD, greater sleep-wake irregularity was associated with lower light exposure during M10 (*r*_42_ = −0.53; *P* < .001) or daytime from 5 am to 9 pm (*r*_42_ = −0.53; *P* < .001) (Figure 2A) but not with nighttime light exposure from 9 pm to 5 am (*r*_42_ = −0.18; *P* = .27) or timing of light exposure (*r*_42_ = −0.22; *P* = .16). The associations remained significant for participants with MOUD treatment only (M10, *r*_33_ = −0.56, *P* < .001; daytime, *r*_33_ = −0.57, *P* < .001). No significant associations between light exposure and sleep-wake irregularity were found in the HC group (M10, *r*_31_ = −0.04, *P* = .83; daytime, *r*_31_ = 0.04, *P* = .84, nighttime, *r*_31_ = −0.31, *P* = .09; MLiT^500^, *r*_31_ = 0.18, *P* = .34). Among participants receiving MOUD, sleep-wake irregularity was associated with years of heroin use after adjusting both for age (partial *r*_26_ = 0.45; *P* = .02) (Figure 2B).

### Brain State Dynamics and Their Associations With Sleep-Wake Irregularity

Among 73 participants, 29 with OUD and 31 in the HC group underwent rfMRI. The mean (SD) number of days between the rfMRI scan and actigraphy data collection was 1.5 (48.8) days.

**Figure 3.**
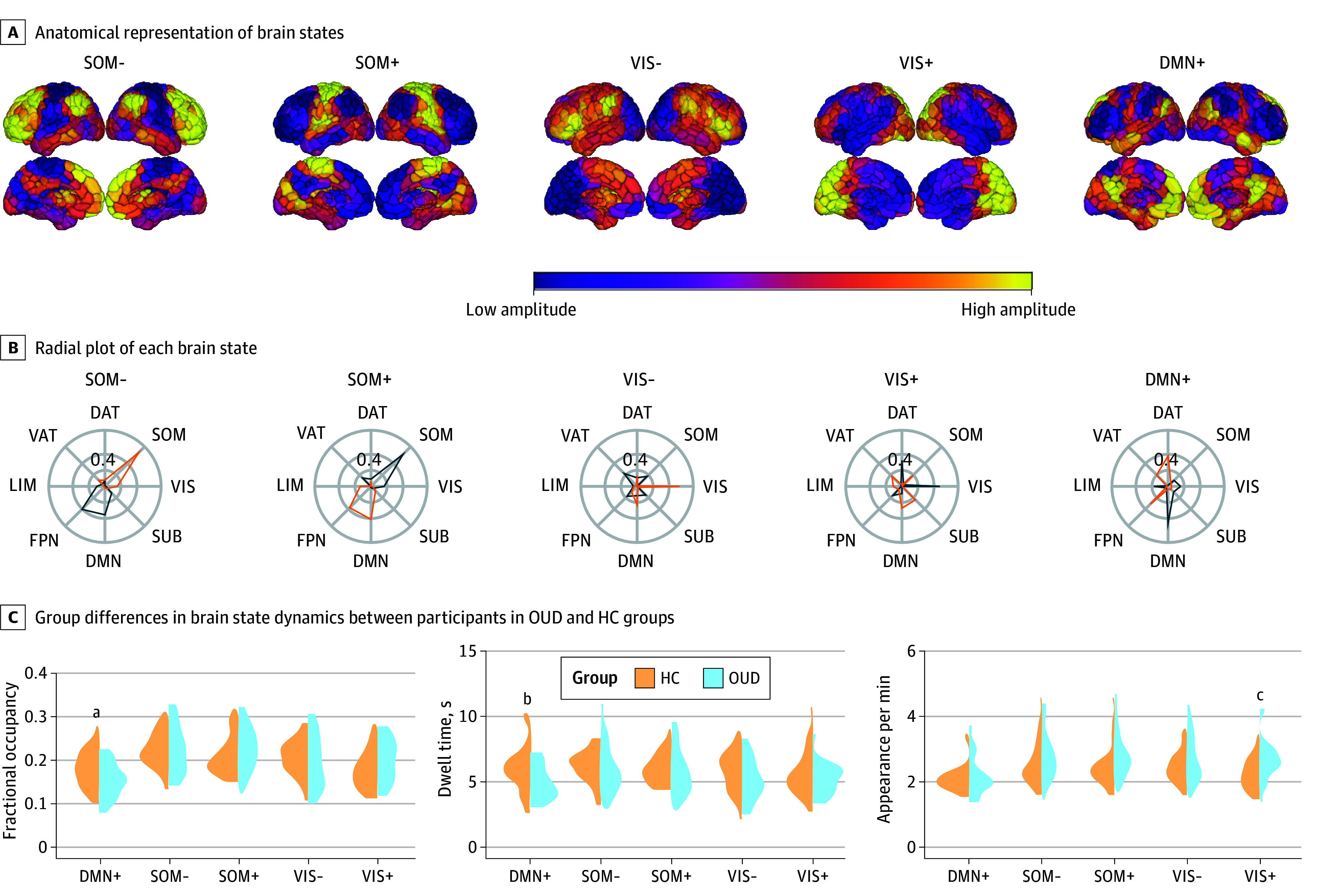
Recurrent Brain States and Their Temporal Dynamics in Opioid Use Disorder (OUD) A, Centroids mapped to colors of the corresponding 454 regions of the Schaefer atlas consisting of 400 cortical and 54 subcortical regions. Each brain state label reflects resting-state functional networks with the most overall similarity. B, Radial plot of each brain state represents cosine similarity of its high-amplitude (blue) and low-amplitude activity (orange) with resting-state functional networks. The outer circle boundary represents a cosine similarity of 0.6; the middle circle, 0.4; and the inner circle, 0.2. C, Fractional occupancy defined as the proportion of repetition times assigned to each brain state. Dwell time calculated by averaging length of time spent in a brain state. Appearance rate determined by the total number of times a state appeared per minute. + and − represent activity above or below regional means, respectively; DAT, dorsal attention network; DMN, default mode network; FPN, frontoparietal network; HC, healthy control group; LIM, limbic network; SOM, somatomotor network; VAT, ventral attention network; VIS, visual network; SUB, subcortical regions. *P* values from 2-sided independent *t* tests. ^a^*P* = .03. ^b^*P* = .02. ^c^*P* = .01.

Based on the predominance of the network patterns in each brain state, the identified brain states were labeled as somatomotor, visual, or DMN-dominated brain states, with activity above or below regional means reported (Figure 3A and 3B). There was high similarity in brain states between participants with OUD and participants in the HC group (ie, coactivation patterns) (eFigure 4 in Supplement 1). In terms of brain state dynamics, participants with OUD showed lower fractional occupancy (*t*_52_ = 2.23; *P* = .03, BH-corrected *P* = .15; Cohen *d* = 0.61) and shorter dwell time (*t*_52_ = 2.35; *P* = .02, BH-corrected *P* = .11; Cohen d = 0.65) in the DMN-dominated brain state with activity above regional means, whereas they had higher appearance rates for the visual network–dominated brain state with activity above regional means (*t*_52_ = 2.68; *P* = .01, BH-corrected *P* = .05; Cohen *d* = 0.74) (Figure 3C). One-way analysis of variance revealed that compared with participants in the HC group, participants receiving MOUD exhibited shorter dwell times for the DMN-dominated brain state with activity above regional means (*F*_2,51_ = 3.28, *P* = .046, BH-corrected *P* = .23; η^2^ = 0.11; post hoc Tukey HSD for the group with MOUD vs HC group, *P* = .04), and participants without MOUD exhibited higher appearance rates for visual network–dominated state with activity above regional means (*F*_2,51_ = 4.17; *P* = .02, BH-corrected *P* = .11; η^2^ = 0.14; post hoc Tukey HSD for group without MOUD vs HC group, *P* = .04). Observed group differences in brain state dynamics were not associated with head motion of the time points included in the analyses in participants with OUD (DMN-dominated brain state with activity above regional means fractional occupancy, *r*_23_ = 0.19, *P* = .38; DMN-dominated brain state with activity above regional means dwell time, *r*_23_ = −0.12, *P* = .60; visual network–dominated brain state with activity above regional means appearance rates, *r*_23_ = 0.37; *P* = .09). Patients treated with methadone vs buprenorphine did not significantly differ in any of these brain state dynamics measures (DMN-dominated brain state with activity above regional means fractional occupancy, *t*_14_ = 1.16, *P* = .27; DMN-dominated brain state with activity above regional means dwell time *t*_14_ = 1.33, *P* = .20; visual network–dominated with activity above regional means appearance rates, *t*_14_ = −0.45; *P* = .66).

Among participants with OUD, greater sleep-wake irregularity was associated with lower fractional occupancy in the DMN-dominated brain state with activity above regional means (*r*_23_ = −0.55; *P* = .007) (Figure 2C). The results remained consistent when we included only 18 participants with OUD who had rfMRI and actigraphy data collected within 30 days (*r*_18_ = −0.52; *P* = .03).

## Discussion

The results of the present cross-sectional study suggested heightened sleep-wake irregularity in individuals with OUD. Notably, among participants with OUD, greater sleep-wake irregularity was associated with longer drug use history as well as lower exposure to light. Compared with HCs, individuals with OUD demonstrated lower fractional occupancy in the brain state dominated by DMN activity, with individuals experiencing more pronounced sleep-wake irregularities displaying exacerbated impairments.

Detrimental outcomes have been associated with greater sleep irregularity. Notably, sleep irregularity has been associated with greater depression in older adults and poorer academic performance in young adults.^17,18^ Among patients with alcohol use disorder who were enrolled in an inpatient alcohol treatment program, sleep irregularity was associated with mood disorders and improved during 3 weeks of treatment.^19^ During the 28-day follow-up after being discharged from inpatient treatment, sleep irregularity was greater among patients who relapsed than among patients who did not.^20^ The findings that negative outcomes associated with sleep irregularity were observed both in individuals who were less affected by fixed work or social schedules (eg, older adults and university students) and in persons living in controlled environments (eg, inpatient setting) suggest potential neurobiological contributions to sleep irregularity. Our findings also showed that sleep-wake irregularity in OUD was not associated with employment status. Additionally, no alterations in sleep duration, physical activity, and phase time were found in participants with OUD. This result is likely to pertain to individuals with OUD who are receiving MOUD or who are in recovery, as prior studies have reported shorter objective sleep duration in persons with active OUD.^21^ In our previous work, we observed that in healthy participants, late phase timing and physical activity were associated with dopamine D_1_ and D_2_ receptor availability, respectively.^8^ Our ongoing studies are now examining whether opioid agonist treatment normalizes dopaminergic receptor levels in OUD. Future studies should apply longitudinal study designs to examine how different components of RAR vary from acute detoxification to extended abstinence and recovery and to evaluate which RAR biomarkers are sensitive to different addiction stages.

Sleep-wake irregularity has been associated with low light exposure in older adults^17^ and in patients with schizophrenia.^22^ Although participants with OUD did not significantly differ from participants in the HC group in light exposure in the present study, greater daytime light exposure was associated with reduced sleep-wake irregularity among participants with OUD. Participants without MOUD showed higher levels of light exposure than both participants with MOUD treatment and in the HC group and exhibited sleep-wake regularity similar to persons in the HC group. This suggests that compared with persons in the HC group, stronger zeitgebers such as light may be required to stabilize the sleep-wake cycle in individuals with OUD. Sleep irregularity may reflect reduced robustness of the endogenous circadian rhythm resulting from disruption of input to the suprachiasmatic nucleus (circadian clock), such as retinal and optic pathology, dysfunction of this nucleus or its outputs, such as melatonin secretion.^23^ Indeed, in animal studies, fentanyl attenuates the effect of light on circadian rhythms^24^ and melatonin decreases fentanyl-induced sleep-wake irregularity.^25^ Sleep-wake regularity in the control group was not associated with light exposure or brain state dynamics, possibly due to a ceiling effect. The robust circadian clock in healthy persons may allow them to optimize their sleep-wake cycle even with moderate light exposure to best support brain functions.

Sleep-wake irregularity was associated with age-independent drug use history and with more severe impairments in DMN-dominated brain state dynamics in participants with OUD. Our finding is in line with a previous study in healthy adolescents and young adults showing that sleep-wake regularity is associated with enhanced DMN functions reflected by a more efficient network structure.^26^ The DMN is involved in multiple functions, including self-related thoughts,^27^ and its impairment has been associated with the cognitive and emotional dysfunctions that emerge at different stages of addiction.^28^ In the present study, sleep-wake irregularity was associated with lower prevalence in the brain state characterized by high activity in the DMN. A previous study revealed a brain coactivation pattern in patients in various states of unconsciousness similar to that which we observed in individuals with OUD.^29^ This similarity suggests that DMN impairments in participants with OUD may interfere with consciousness, self-awareness, and ability to flexibly shift attention between internal and external worlds.^11^

### Limitations

This study has limitations. We did not observe differences in RAR or brain state dynamics between individuals receiving methadone vs buprenorphine, both of which are opioid agonist medications, albeit with different efficacies. We found that participants without MOUD showed less sleep irregularity than participants with MOUD treatment, although the small sample size of participants without MOUD limits our conclusions. It remains unclear whether these differences were associated with opioid agonist medications’ effects on melatonin secretion^30^ and light sensitivity,^24^ preexisting differences in an individual’s capability to recover from addiction, or lifestyle, as participants receiving no MOUD showed the highest levels of light exposure in this study. Due to the observational nature of our study, no causality of the association between sleep irregularity and DMN functions can be established. To examine the directionality, intervention studies that regulate sleep-wake cycle or modulate DMN functions will be required. A small number of participants with MOUD treatment used heroin, cocaine, or cannabis in the last 30 days, which confounds the interpretation of our findings. Although sleep-wake regularity did not differ between persons with MOUD treatment who used drugs (heroin, cocaine, or cannabis) vs who did not, nor did it correlate with the number of days of using heroin, cocaine or cannabis in the last 30 days among users, the associations with recent drug use need to be further clarified in a larger and well-powered study.

## Conclusions

This cross-sectional study found sleep-wake irregularity among participants receiving MOUD that was associated with their drug use history and with disrupted DMN functions. Additionally, preliminary evidence suggests that extra light exposure may help regulate the sleep-wake cycle in individuals with OUD, facilitating brain recovery. The current study advances our understanding of the neurobiological underpinnings of sleep-wake irregularity in persons with OUD and suggests that interventions improving sleep-wake irregularity may be beneficial for individuals receiving MOUD.

## Notes

Supplement 1. **eAppendix 1.** Actigraphy analysis **eAppendix 2.** 21 RAR variables and their definitions **eAppendix 3.** Non-wear periods in actigraphy data **eAppendix 4.** MRI acquisition **eAppendix 5.** MRI preprocessing **eAppendix 6.** Analysis of brain states and dynamics **eAppendix 7.** Power analysis **eAppendix 8.** Effects of length of opioid agonist treatment, acute drug use, pandemic and employment status on RAR **eAppendix 9.** The effect of daylength on light exposure **eFigure 1.** Choosing K **eFigure 2.** RAR components in OUD vs HC (independent *t*-tests) **eFigure 3.** Light exposures in OUD vs HC (independent *t*-tests) **eFigure 4.** Comparison of OUD vs HC brain states **eTable.** Principal component analysis results for 21 RAR variables
eReferences.

Supplement 2.
Data Sharing Statement
