# Supplementary material for: Rest-Activity Rhythms, Their Modulators, and Brain-Clinical Correlates in Opioid Use Disorder
Source: JAMA Netw Open. 2025 Feb 4;8(2):e2457976. doi: 10.1001/jamanetworkopen.2024.57976 (PMC11795329; doi:10.1001/jamanetworkopen.2024.57976)
Supplement: Supplement 1. — eAppendix 1. Actigraphy analysis eAppendix 2. 21 RAR variables and their definitions eAppendix 3. Non-wear periods in actigraphy data eAppendix 4. MRI acquisition eAppendix 5. MRI preprocessing eAppendix 6. Analysis of brain states and dynamics eAppendix 7. Power analysis eAppendix 8. Effects of length of opioid agonist treatment, acute drug use, pandemic and employment status on RAR eAppendix 9. The effect of daylength on light exposure eFigure 1. Choosing K eFigure 2. RAR components in OUD vs HC (independent t-tests) eFigure 3. Light exposures in OUD vs HC (independent t-tests) eFigure 4. Comparison of OUD vs HC brain states eTable. Principal component analysis results for 21 RAR variables eReferences. [file jamanetwopen-e2457976-s001.pdf]

Supplementary Online Content

Zhang R, Manza P, Demiral SB, et al. Rest-activity rhythms, their modulators, and brain-clinical correlates in opioid use disorder. *JAMA Netw Open*. 2025;8(2):e2457976. doi:10.1001/jamanetworkopen.2024.57976

eAppendix 1. Actigraphy analysis

eAppendix 2. 21 RAR variables and their definitions

eAppendix 3. Non-wear periods in actigraphy data

eAppendix 4. MRI acquisition

eAppendix 5. MRI preprocessing

eAppendix 6. Analysis of brain states and dynamics

eAppendix 7. Power analysis

eAppendix 8. Effects of length of opioid agonist treatment, acute drug use, pandemic and employment status on RAR

eAppendix 9. The effect of daylength on light exposure

eFigure 1. Choosing K

eFigure 2. RAR components in OUD vs HC (independent t-tests)

eFigure 3. Light exposures in OUD vs HC (independent t-tests)

eFigure 4. Comparison of OUD vs HC brain states

eTable. Principal component analysis results for 21 RAR variables

eReferences.

© 2025 Zhang R et al. *JAMA Network Open*.

This supplementary material has been provided by the authors to give readers additional information about their work.

© 2025 Zhang R et al. *JAMA Network Open*.

eAppendix 1. Actigraphy analysis

For actigraphy analyses, we utilized both parametric and nonparametric measures as they offer complimentary insights. While parametric measures capture the size, timing and shape of rhythms [1], nonparametric measures are based on raw data counts and do not rely on a priori assumptions about the waveform e.g., a cosine shape of activity data [2]. We processed the raw accelerometer data in .bin format for nonparametric analysis using the R package GGIR (v3.0-6)[3,4]. GGIR employs a heuristic algorithm based on changes in the z-angle to detect sleep periods, defined as sustained inactivity bouts and has shown high accuracy without sleep diaries[5]. For parametric (extended cosinor) analysis, GGIR uses R package ActCR as a dependency and acceleration values were averaged per minute and then log-transformed. In total, 21 RAR variables from both parametric and nonparametric modeling were calculated.

© 2025 Zhang R et al. *JAMA Network Open*.

eAppendix 2. 21 RAR variables and their definitions

Parametric measures: **alpha** (narrower active period with higher values), **acrophase** (time of peak activity level), **amplitude** (peak-nadir difference), **up-mesor** (time when activity passes up through mesor, approximately the time of increasing activity in the morning), **down-mesor** (time when activity passes down through mesor, approximately the time of settling down for the night), **pseudo-F statistic** (how well the obtained rest-activity data fitted the 24-h rhythm model; lower F values indicate poorer model fit and greater rhythm irregularity). Non-parametric measures: **M10** (the 10-hour period of maximum activity, roughly daytime activity level), **M10hr** (starting time of M10), **L5** (the 5-hour period of minimum activity, higher value indicating less restful sleep), **L5hr** (L5 starting time), **daily mean activity** (mean activity across 24 hours), **sleep onset**, **wakeup time**, **sleep duration** and **their day-to-day variations (i.e., Standard deviations)**, **Intra-daily variability** (IV; the variations of rest-activity rhythm within each 24-hour period), **Inter-daily stability** (IS; the similarity of one 24-hour period to the next), **Sleep regularity index** (the likelihood that an individual is in the same behavior state i.e., asleep or awake, at any two time points 24 hours apart, and was calculated per day pair) and **its standard deviation**.

© 2025 Zhang R et al. *JAMA Network Open*.

eAppendix 3. Non-wear periods in actigraphy data

In GGIR, non-wear time is detected when both of the following criteria are met for at least two out of the three accelerometer axes: 1) the standard deviation of the accelerations is less than reference values, 2) the range of accelerations is less than 50 mg.

Detected non-wear time for HC was 3.08% (SD: 7.92), for MOUD+ was 2.07% (SD: 2.76), for MOUD-was 3.59% (SD: 8.46). No group differences were found ($F_{2,70}$=.32, p=.727).

We only included days when valid hours were greater than 16. Number of valid days for HC was 6.25 days (SD: 1.15), for MOUD+ was 6.30 days (SD: .59), for MOUD- was 6.67 days (SD: .50). No group differences were found ($F_{2,70}$=.80, p=.455).

For non-parametric analyses, for each non-wear time point, imputation was applied by taking the average of all valid data (monitor worn) on similar time points on other days of the measurement. *For cosinor analyses (parametric analyses),* invalid data points such as caused by non-wear were set to missing (NA) to prevent the imputation approach used elsewhere in GGIR to influence the cosinor analysis.

© 2025 Zhang R et al. *JAMA Network Open*.

eAppendix 4. MRI acquisition

Participants were scanned on a 3T Magnetom Prisma scanner (Siemens Medical Solutions USA, Inc., Malvern, PA) with a 32-channel head coil. rfMRI data were collected over an 8-min period using a multi-echo, multiband echo-planar imaging (EPI) sequence (Multiband factor=3, TR=891 ms, TE=16, 33 and 48 ms, FA=57°, 45 slices with 2.9 x 2.9 x 3.0 mm voxels, 520 time points). Multi-echo images were combined for each time point using an echo-time weighted average of TEs. During scanning, a fixation cross was presented on a black background using an LCD monitor and participants were instructed to keep their eyes open. T1-weighted 3D MPRAGE (TR/TE=2400/2.24 ms, FA=8°) was used to acquire high-resolution anatomical brain images with 0.8 mm isotropic voxels FOV=240 x 256 mm.

eAppendix 5. MRI preprocessing

The data were preprocessed using CONN toolbox 21a [6] including rigid body realignment, spatial normalization to MNI space, smoothing (FWHM=6mm), band-pass filtering (0.01-0.08 Hz), linear detrending, head motion regression (3 rotational, 3 translational and their derivatives), removal of signals within the CSF and the WM using aCompcor, a method for identifying principal components associated with segmented WM and CSF. Using custom MATLAB code, we further scrubbed volumes with a framewise displacement (FD) threshold of 0.25 mm. We excluded 6 OUD participants, who had a mean FD > 0.6 mm before scrubbing or the number of time frames less than 180 after scrubbing from the analyses.

© 2025 Zhang R et al. *JAMA Network Open*.

eAppendix 6. Analysis of brain states and dynamics

To identify brain states i.e., brain co-activation patterns, we first parcellated denoised voxel-level data into a 454-node Schaefer atlas (400 cortical regions and 54 subcortical regions) [7,8] followed by demeaning each ROI time-series. Subsequently, we concatenated demeaned ROI timeseries from all participants into a matrix (row: $N_{Subjects}$*$N_{time\ points}$; column: N_{ROIs}), upon which we applied k-means clustering [9]. This approach allowed us to investigate brain dynamics at a maximum temporal resolution of 1 TR. We performed k-means clustering for k=2-22, where k was the number of clusters (k^2 must be less than the number of TRs to capture all transitions) using Pearson correlation as the distance metric. The process was iterated 50 times with random initializations, and the solution with the optimal data separation was chosen. The optimal number of clusters (k) was determined based on incremental variance explained by the lowest error solution at each value of k. We chose k=5 because additional variance explained by increasing k beyond k=5 was less than 1%. (**Figure S1**). To further ensure the reliability of our partitions, we independently repeated the process 10 times and computed adjusted mutual information between each of the 10 resulting partitions. The partition demonstrating the highest adjusted mutual information with all other partitions was selected for further analysis [10]. The clusters were defined as brain states and labeled by assessing the cosine similarity of their centroid's positive and negative activations with a binary presentation of seven a priori-defined cortical brain functional networks [11] plus a network with 54 subcortical regions. Positive values of cluster centroid reflect activations above mean (high amplitude), while negative values reflect activations below mean (low amplitude).

Following this, we then analyzed the dynamic characteristics of the identified brain states. Fractional occupancy was defined as the proportion of TRs assigned to each brain state. Dwell

time was calculated by averaging length of time (number of contiguous TRs *TR) spent in a brain state. Appearance rate was determined by the total number of times a state appeared per minute.

© 2025 Zhang R et al. *JAMA Network Open*.

eAppendix 7. Power analysis (sensitivity)

Based on previous study, comparing various objective sleep parameters and subjective sleep between MOUD and HC[12–14], a medium to large effect size was observed (objective: Cohen's d=0.77-1.77; subjective: 2.40-3.15). The effect size of circadian alteration in abstinent heroin users was medium to large (Cohen's d=.52-1.30)[15]. The current sample size of N=31 HC and N=42 OUD will be able to detect a medium effect d=0.67 (power=.8, a=.05).

© 2025 Zhang R et al. *JAMA Network Open*.

eAppendix 8. Effects of length of opioid agonist treatment, acute drug use, pandemic and employment status on RAR

Effects of length of opioid agonist treatment

Days on opioid agonist treatment were not associated with sleep-wake irregularity after controlling for age among MOUD+ (r_{23}=.33, p=.11).

Effects of acute drug use

8 MOUD+ who used heroin in the past 30 days, did not differ from 25 MOUD+ who did not use heroin recently in sleep-wake regularity (t_{31}=1.61, p=.12). Among the 8 MOUD+, days of heroin use in the last 30 days was not associated with sleep-wake irregularity either (r_8=.17, p=.69). The same approach was applied to examine the effect of recent cannabis (n=12 MOUD+) and cocaine use (n=6 MOUD+). No significant results were found (cannabis: t_{30}= -.11, p=.91; r_{12}=.39, p=.21; cocaine: t_{31}= 1.31, p=.20; r_6=.39, p=.45).

Effects of the pandemic

The effect of the pandemic on RAR components was not significant (late phase timing $F_{2,70}$=.973, p=.38; sleep-wake irregularity $F_{2,70}$=1.821, p=.17; physical activity $F_{2,70}$=1.459, p=.24; long and restful sleep $F_{2,70}$=1.025, p=.36).

Effects of employment status

Work schedules can influence sleep-wake patterns and N=41 OUD participants reported their employment status (24 employed and 17 unemployed) during clinical interviews. In OUD,

© 2025 Zhang R et al. *JAMA Network Open*.

employment status did not significantly contribute to their sleep-wake irregularity (t_{39}=1.40, p=.17).

© 2025 Zhang R et al. *JAMA Network Open*.

eAppendix 9. The effect of daylength on light exposure

Daylength was calculated as the daytime plus civil twilight on the study days using R package "suncalc", where calculations were based on geographic location of the study locations: Bethesda, Mayland, USA: Latitude=39.00, Longitude=-77.10. Daylength was positively associated with daytime light exposure from 5 am to 9 pm ($r=.60$, $p<.001$) but not nighttime light exposure from 9 pm to 5 am the next day ($r=.09$, $p=.45$). However, daylength did not differ between groups (MOUD+, MOUD- VS HC) ($F_{2,70}$=.1.24, $p=.30$). Thus, it's less likely that our findings result from seasonal effect.

© 2025 Zhang R et al. *JAMA Network Open*.

eFigure 1. Choosing K

Gained variance explained when k increases from k-1 to k. When k>5, less than 1% of additional variance is explained.

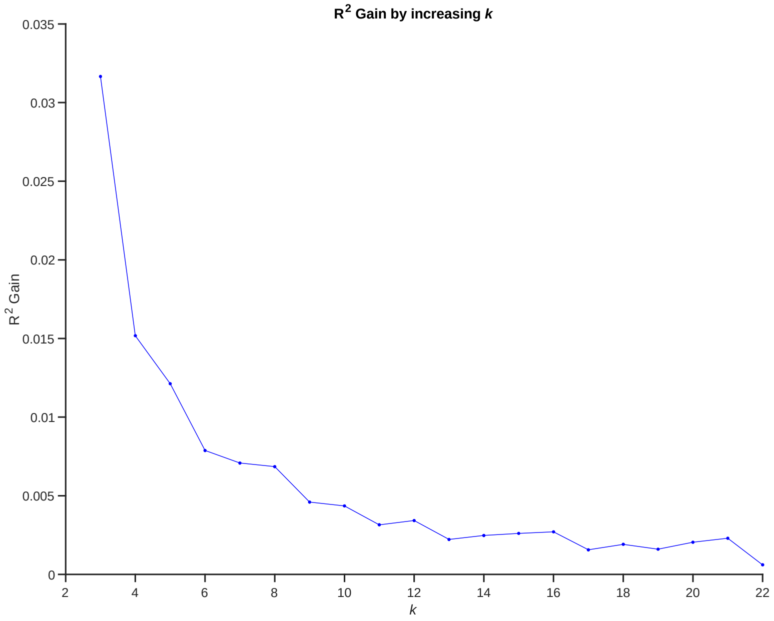

© 2025 Zhang R et al. *JAMA Network Open*.

eFigure 2. RAR components in OUD vs HC (independent t-tests)

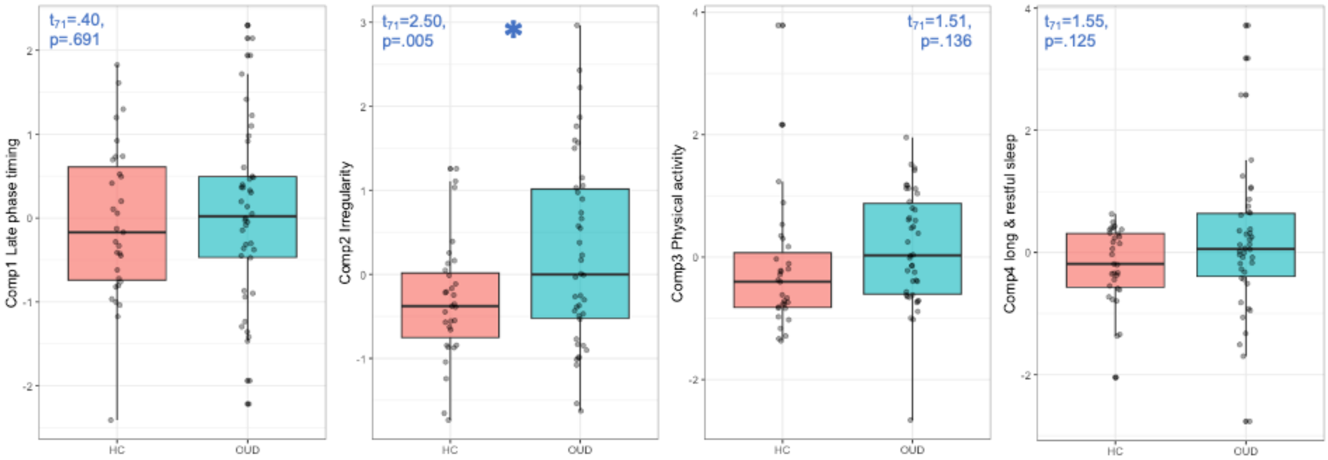

© 2025 Zhang R et al. *JAMA Network Open*.

eFigure 3. Light exposures in OUD vs HC (independent t-tests)

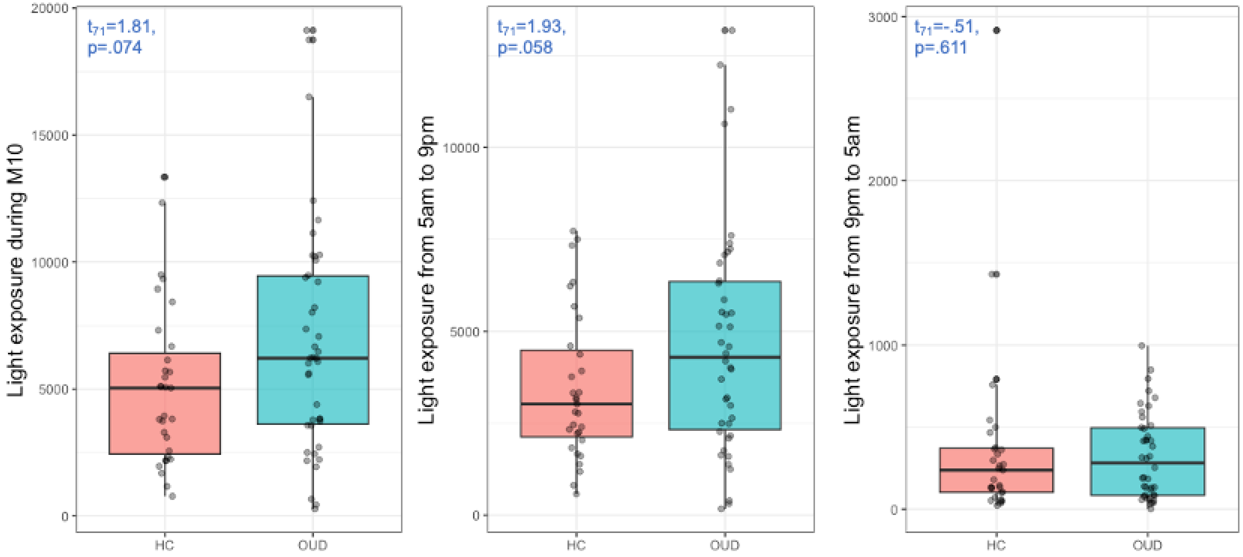

© 2025 Zhang R et al. *JAMA Network Open*.

eFigure 4. Comparison of OUD vs HC brain states

(A) Cluster centroids in OUD vs HC

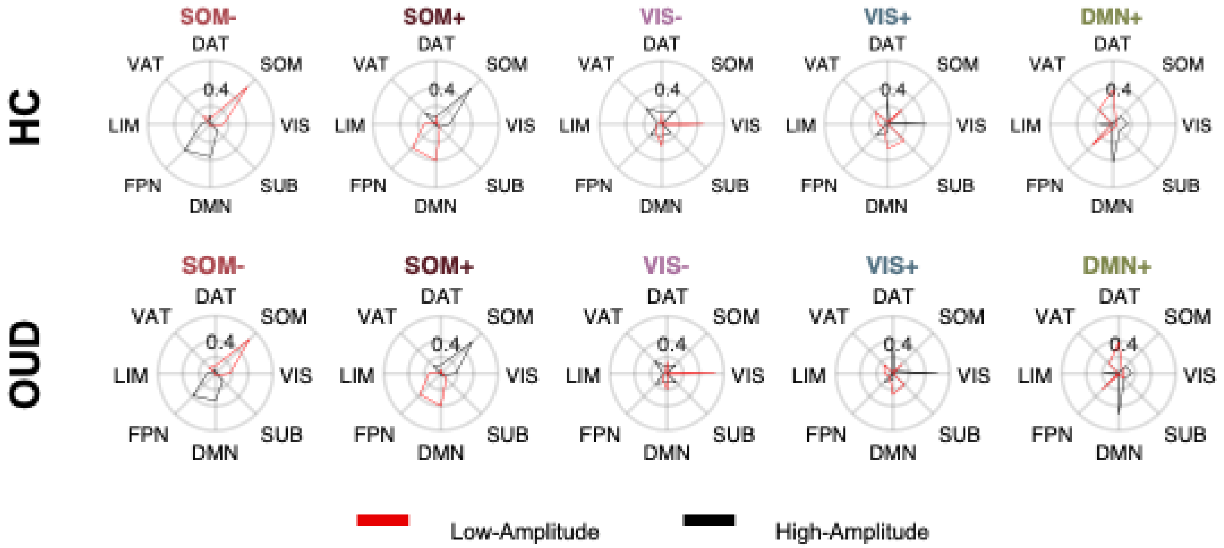

(B) Correlation between the centroids of OUD and HC participants showing high consistency (diagonal): r= .93-.96

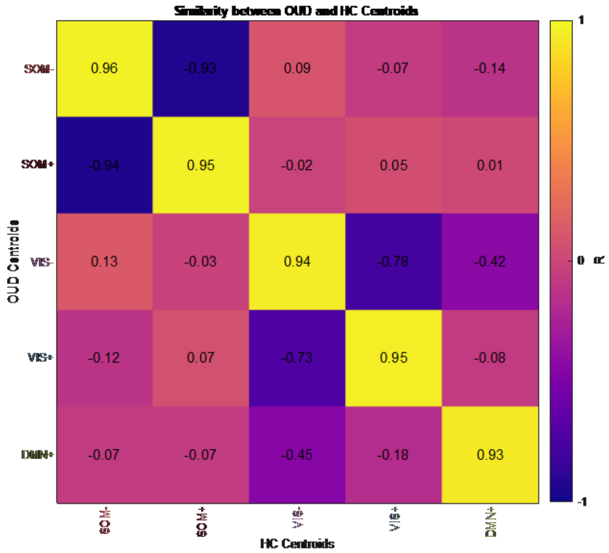

© 2025 Zhang R et al. *JAMA Network Open*.

eTable. Principal component analysis results for 21 RAR variables

Rotated Component Matrix	Component			
	1	2	3	4
IS_interdailystability		-.605		
IV_intradailyvariability			-.680	
amplitude			.590	
alpha				.638
acrophase	.922			
UpMesor	.655			
DownMesor				
F_pseudo				
L5hr	.893			
L5				-.523
M10hr	.840			
M10			.853	
Daily mean physical activity			.861	
Sleep duration				.786
Sleep duration variation		.704		
Sleep onset	.803			
Sleep onset variation		.777		
Wakeup time	.845			
Wake up time variation		.804		
SleepRegularityIndex		-.775		
SleepRegularityIndex variation		.611		

Extraction Method: Principal Component Analysis.
Rotation Method: Varimax with Kaiser Normalization.
Coefficient values greater than 0.5 are listed.

© 2025 Zhang R et al. *JAMA Network Open*.

eReferences.

1. Marler MR, Gehrman P, Martin JL, Ancoli-Israel S. The sigmoidally transformed cosine curve: a mathematical model for circadian rhythms with symmetric non-sinusoidal shapes. *Stat Med*. 2006;25(22):3893-3904. doi:10.1002/sim.2466

2. van Someren EJW, Hagebeuk EEO, Lijzenga C, et al. Circadian rest-activity rhythm disturbances in alzheimer's disease. *Biological Psychiatry*. 1996;40(4):259-270. doi:10.1016/0006-3223(95)00370-3

3. van Hees VT, Fang Z, Langford J, et al. Autocalibration of accelerometer data for free-living physical activity assessment using local gravity and temperature: an evaluation on four continents. *Journal of Applied Physiology*. 2014;117(7):738-744. doi:10.1152/japplphysiol.00421.2014

4. van Hees VT, Sabia S, Anderson KN, et al. A Novel, Open Access Method to Assess Sleep Duration Using a Wrist-Worn Accelerometer. *PLoS ONE*. 2015;10(11):e0142533. doi:10.1371/journal.pone.0142533

5. van Hees VT, Sabia S, Jones SE, et al. Estimating sleep parameters using an accelerometer without sleep diary. *Sci Rep*. 2018;8. doi:10.1038/s41598-018-31266-z

6. Whitfield-Gabrieli S, Nieto-Castanon A. Conn: a functional connectivity toolbox for correlated and anticorrelated brain networks. *Brain Connect*. 2012;2(3):125-141. doi:10.1089/brain.2012.0073

7. Schaefer A, Kong R, Gordon EM, et al. Local-Global Parcellation of the Human Cerebral Cortex from Intrinsic Functional Connectivity MRI. *Cerebral Cortex*. 2018;28(9):3095-3114. doi:10.1093/cercor/bhx179

8. Tian Y, Margulies DS, Breakspear M, Zalesky A. Topographic organization of the human subcortex unveiled with functional connectivity gradients. *Nat Neurosci*. 2020;23(11):1421-1432. doi:10.1038/s41593-020-00711-6

9. Cornblath EJ, Ashourvan A, Kim JZ, et al. Temporal sequences of brain activity at rest are constrained by white matter structure and modulated by cognitive demands. *Commun Biol*. 2020;3(1):1-12. doi:10.1038/s42003-020-0961-x

10. Singleton SP, Luppi AI, Carhart-Harris RL, et al. Receptor-informed network control theory links LSD and psilocybin to a flattening of the brain's control energy landscape. *Nat Commun*. 2022;13(1):5812. doi:10.1038/s41467-022-33578-1

11. Yeo BTT, Krienen FM, Sepulcre J, et al. The organization of the human cerebral cortex estimated by intrinsic functional connectivity. *J Neurophysiol*. 2011;106(3):1125-1165. doi:10.1152/jn.00338.2011

© 2025 Zhang R et al. *JAMA Network Open*.

12. Xiao L, Tang Y lang, Smith AK, et al. Nocturnal sleep architecture disturbances in early methadone treatment patients. *Psychiatry Research*. 2010;179(1):91-95. doi:10.1016/j.psychres.2009.02.003

13. Orr WC, Stahl ML. Sleep Patterns in Human Methadone Addiction. *British Journal of Addiction to Alcohol & Other Drugs*. 1978;73(3):311-315. doi:https://doi.org/10.1111/j.1360-0443.1978.tb00158.x

14. Mehtry V, Nizamie SH, Parvez N, Pradhan N. Sleep profile in opioid dependence: a polysomnographic case-control study. *J Clin Neurophysiol*. 2014;31(6):517-522. doi:10.1097/WNP.0000000000000117

15. Li S xia, Shi J, Epstein DH, et al. Circadian alteration in neurobiology during 30 days of abstinence in heroin users. *Biol Psychiatry*. 2009;65(10):905-912. doi:10.1016/j.biopsych.2008.11.025

© 2025 Zhang R et al. *JAMA Network Open*.
