# Supplementary material for: Rest-Activity Rhythms, Their Modulators, and Brain-Clinical Correlates in Opioid Use Disorder
Source: JAMA Netw Open. 2025 Feb 4;8(2):e2457976. doi: 10.1001/jamanetworkopen.2024.57976 (PMC11795329; doi:10.1001/jamanetworkopen.2024.57976)
Supplement: Supplement 2. — Data Sharing Statement [file jamanetwopen-e2457976-s002.pdf]

Data Sharing Statement

Zhang. Rest-Activity Rhythms, Their Modulators, and Brain-Clinical Correlates in Opioid Use Disorder. *JAMA Netw Open*. Published February 04, 2025.
doi:10.1001/jamanetworkopen.2024.57976

Data
Data available: Yes
Data types: Deidentified participant data
How to access data: rui.zhang@nih.gov
When available: With publication

Supporting Documents
Document types: None

Additional Information
Who can access the data: researchers whose proposed use of the data has been approved
Types of analyses: for a specified purpose
Mechanisms of data availability: with a signed data access agreement
